# Identification of Key microRNAs and Genes in Infantile Hemangiomas

**DOI:** 10.3389/fgene.2022.766561

**Published:** 2022-03-11

**Authors:** Cong Fu, Kun Yang, Yuqing Zou, Ran Huo

**Affiliations:** ^1^ Department of Burn and Plastic Surgery, Shandong Provincial Hospital, Shandong First Medical University, Jinan, China; ^2^ Department of Medicine, Shandong University, Jinan, China

**Keywords:** infantile hemangiomas, micrornas, hub genes, bioinformatics, angiogenesis

## Abstract

Infantile hemangiomas (IHs) are the most frequent vascular tumors that occur during infancy. Microribonucleic acids (miRNAs) have been demonstrated as critical regulators of gene expression in various diseases. However, the function of miRNAs in IH still remains largely unknown. In the present study, we performed a miRNA microarray analysis of IH and identified 68 differentially expressed miRNAs (DEMs). In addition, miRNA-gene networks and protein-protein interactions were constructed, and the hub miRNAs and genes of IH were screened out. Gene Ontology (GO) and Kyoto Encyclopedia of Genes and Genomes (KEGG) pathway analysis were used for biological analysis of DEMs and differentially expressed genes (DEGs). The pathway enrichment analysis of DEMs revealed several tumor-related pathways, including proteoglycans in cancer, signaling pathway regulating pluripotency of stem cells and TGF-beta signaling pathway. DEGs were mainly enriched in biological processes, including intracellular signal transduction, cell adhesion, and cell death. KEGG pathway analysis indicated that DEGs were enriched in tumorigenesis- and angiogenesis-related pathways such as proteoglycans in cancer, MAPK signaling pathway and Rap1 signaling pathway. Collectively, this study first established a comprehensive miRNA-gene network in IH, which should provide novel insights into IH pathogenesis and be beneficial to the understanding of neovascularization-related disorders.

## Introduction

Infantile hemangiomas (IHs) are the most frequent vascular tumors that occur during infancy and childhood with a high incidence rate of 3–10%. They are more prevalent in females, low-birth weight, and premature infants (Drolet et al., 2008; Chen et al., 2013). IHs typically appear within the first month of life and progress rapidly during infancy, followed by a slow spontaneous involution phase extending from 1 year until five to 7 years of age. As IHs most frequently occur in the facial region, some IHs can be disfiguring, functional impairing, ulcerating, bleeding, or even life-threatening (Vildy et al., 2015; Cazeau et al., 2017).

Proliferative IH consists of massive proliferating capillaries and endothelial cells (Darrow et al., 2015). It has been widely acknowledged that angiogenesis and vasculogenesis both play a critical role in IH pathogenesis (Boscolo and Bischoff, 2009). Several vascular-related signaling pathways have been illuminated in IH-associated mechanisms, including hypoxia-inducible factor 1 (HIF)-mediated, Notch signaling, phosphoinosite 3 kinase (PI3K)/protein kinase B (Akt)/mechanistic target of rapamycin (mTOR), and vascular endothelial growth factor (VEGF/VEGFR) pathways (Ji et al., 2014). However, IH pathogenesis processes still remain largely unknown.

Micro-ribonucleic acids (miRNAs) are small noncoding RNA molecules containing about 22 nucleotides that function in post-transcriptional regulation of gene expression. An extensive number of studies have demonstrated that miRNAs are involved in various biological processes, including tumor angiogenesis and vascular-related disorders (Pourrajab et al., 2015; Mashreghi, 2017). In addition, some miRNAs have been reported as biomarkers of specific diseases (Mariam et al., 2022), especially in tumors (Li et al., 2022; Tu et al., 2022). Nonetheless, there are fewer studies focused on the comprehensive miRNA-gene interaction in IH. We have previously identified mRNA profiling in IH (Liu, 2016). In the present study, we performed a miRNA microarray analysis of IH and first constructed a general miRNA-mRNA network by integration of miRNA and mRNA profiling. In addition, several hub miRNAs and genes were identified and related biological pathways were revealed via bioinformatics. This study will provide novel insights into IH pathogenesis and be beneficial for understanding neovascularization-related disorders.

## Materials and Methods

### Ethics Statement

This study was approved by the Institutional Ethics Review Board of Shandong provincial hospital (No. 2016–309). Informed consents were obtained from the parents of the enrolled patients.

### Patients and Samples

Six samples were collected from October 2016 to June 2017 at the Shandong provincial hospital, including three proliferative IH tissues and three adjacent normal tissues. The diagnosis and phase of IH samples were confirmed by two independent doctors. All samples were immediately immersed in RNALater (Qiagen, Hilden, Germany) overnight at 4°C and preserved at −80°C.

### Microarray Analysis

Total RNA was extracted from frozen samples with TRIzol reagent (Invitrogen, Carlsbad, CA, United States) according to the manufacturer’s instructions. RNA concentration was determined by Nanodrop 2000 (Thermo Scientifc, Waltham, MA, United States). The quality of RNA was measured using an Agilent 2100 bioanalyzer and an associated RNA 6000 Nano and Pico Lab Chip kits (Agilent Technologies, Santa Clara, CA, United States). MiRNA microarray was performed utilizing Gene Chip miRNA 4.0 (Affymetrix, Santa Clara, CA, United States). The complete microarray data were deposited in Gene Expression Omnibus (GEO) database [accession number GSE100682 (NCBI tracking system #18530520)].

### Identification of Differentially Expressed MiRNAs and Integration With Deregulated Genes From Previous Studies

miRNA (GSE100682) profiles were used for identification of differentially expressed miRNAs (DEMs) in IH samples. Raw data from the miRNA microarray were normalized and summarized using the log scale robust multi -array analysis (RMA) method (Bolstad et al., 2003; Irizarry, 2003; Irizarry et al., 2003). The statistical significance was detected by significance analysis of microarrays (SAM) software, version 4.01 (http://statweb.stanford.edu/∼tibs/SAM/), which calculates permutation-based FDR values (Tusher et al., 2001). MiRNAs presenting both |fold changes| >2.0 and false discovery rates (FDR) < 0.05 differentially expressed between IH and control samples were regarded as a significant differential expression. The differential test was performed using SAM. A validated target module of miRWalk 2.0 was used for prediction of target genes of identified deregulated miRNAs (Dweep et al., 2011). The intersection of miRNA targeted and deregulated genes from our previous study (Liu, 2016) were then screened out as the final dataset of DEGs. Finally, DEMs in IH were selected by comparison of DEGs and miRNA-mRNA interactions from miRWalk. The DEM-DEG network was constructed using Cytoscape (Shannon et al., 2003).

### Functional Enrichment Analysis of Differentially Expressed miRNAs-Differentially Expressed Genes Network

Diana mirPath v3.0, a miRNA pathway web-server, was used for pathway enrichment analysis of DEMs (Vlachos et al., 2015). The Database for Annotation, Visualization and Integrated Discovery (DAVID) v6.8, which provides a comprehensive set of function annotation tools of gene list, was used for Gene Ontology (GO) and Kyoto Encyclopedia of Genes and Genomes (KEGG) functional enrichment analysis of DEGs (Huang et al., 2009). *p* < 0.05 was considered statistically significant.

### Protein-Protein Interaction of Differentially Expressed Genes

Search Tool for the Retrieval of Interacting Genes/Proteins (STRING) was used to explore the interaction of DEGs in IH (Szklarczyk et al., 2011). Interactions with combined score ≥0.4 were selected as significant. The protein-protein interaction (PPI) network was depicted by Cytoscape. The plug-in Molecular Complex Detection (MCODE) was utilized to screen modules of the PPI networks in Cytoscape. Modules with MCODE scores >5 and number of nodes >10 were screened out.

## Results

### Identification of Differentially Expressed miRNAs and Differentially Expressed Genes

**FIGURE 1:**
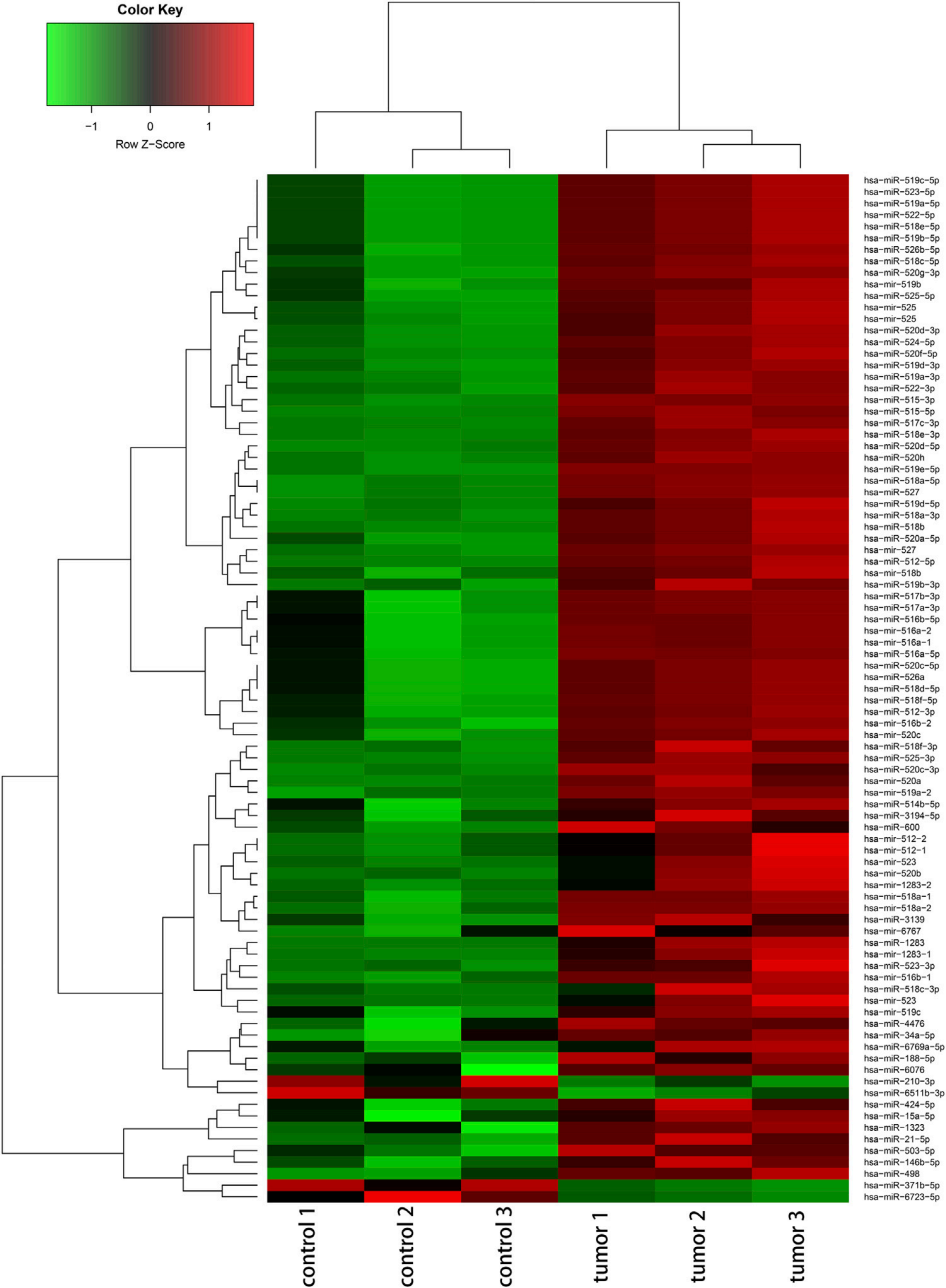
Hierarchical cluster analysis of deregulated miRNAs (|fold change|≥ 2 and FDR <0.05) between IH and controls.

**FIGURE 2:**
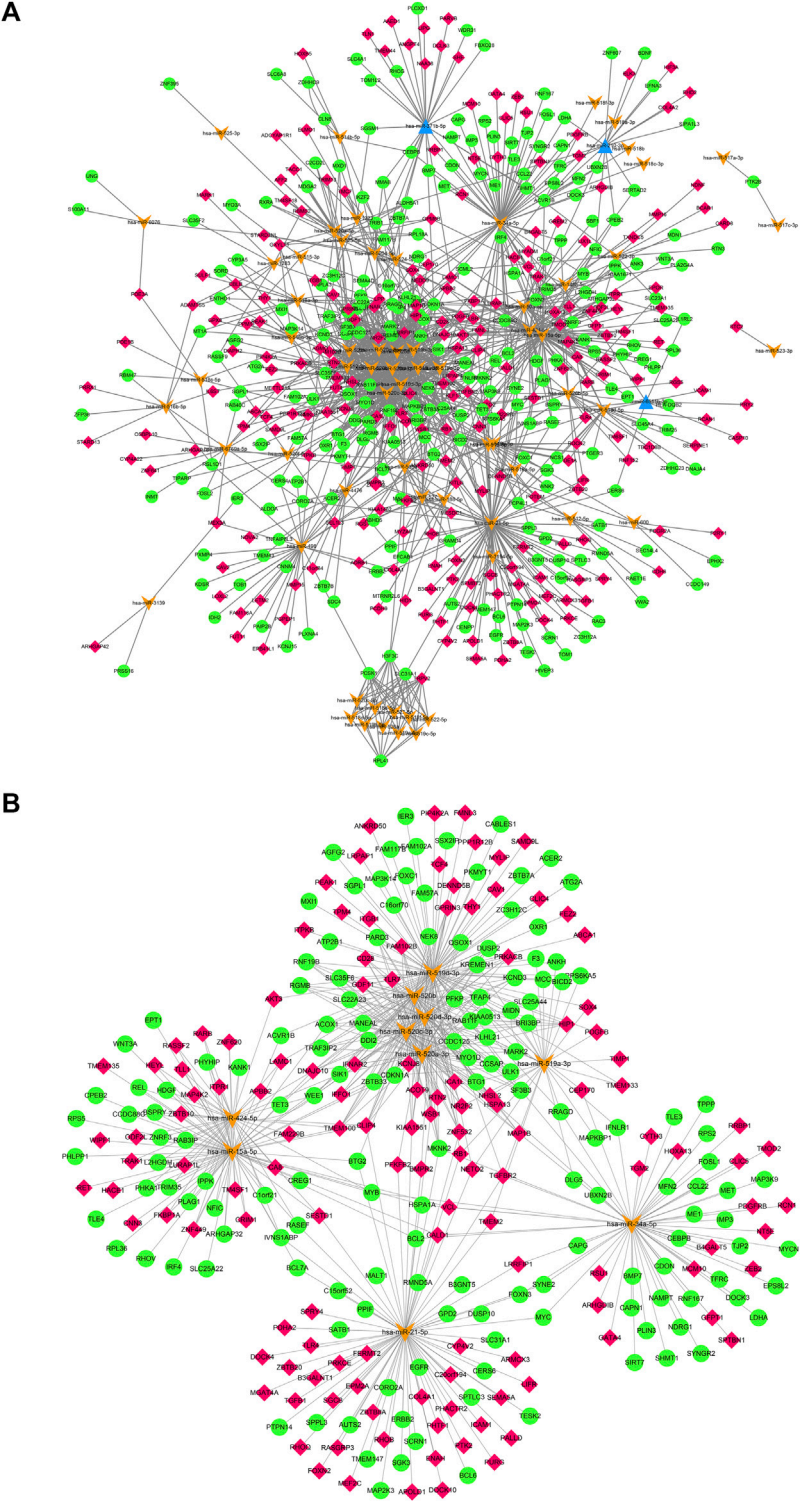
**(A)**. Regulatory network of DEMs and their target DEGs of IH depicted by cytoscape. **(B)**. hub DEM-DEG network of IH. Orange arrows mean up-regulated DEMs, blue triangles mean down-regulated DEMs, red rhombus mean up-regulated DEGs, green cycles mean down-regulated DEGs.

The deregulated miRNAs from proliferative IH and normal skin tissues as identified by microarray are shown as Figure 1. With integrated miRNA and mRNA profiles in IH, a total of 68 DEMs, including 65 up-regulated and three down-regulated miRNAs, and 475 DEGs, including 228 up-regulated and 247 down-regulated genes, were identified from IH and normal skin tissues. The interaction network of DEMs and DEGs are shown in Figure 2A. The top 10 DEMs were selected as hub miRNAs, including miR-519d-3p, miR-21-5p, miR-15a-5p, miR-34a-5p, miR-520a-3p, miR-520b, miR-520c-3p, miR-520d-3p, miR-424-5p, and miR-519a-3p, based on the degree of interactions. The hub DEM-DEG network is shown in Figure 2B.

### Pathway Enrichment Analysis of Differentially Expressed miRNAs

**FIGURE 3:**
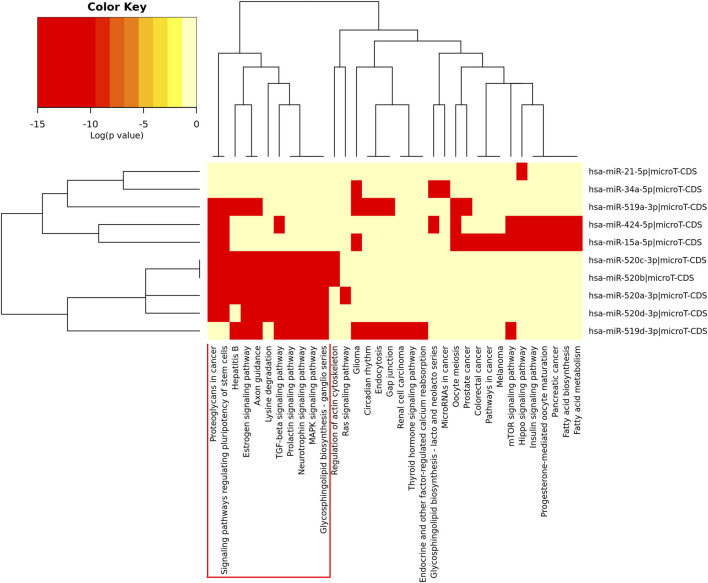
Heatmap of pathway enrichment analysis of the hub DEMs.

The heatmap of pathway analysis showed hub DEMs were mainly involved in proteoglycans in cancer, estrogen signaling pathway, TGF-beta signaling pathway, signaling pathways regulating pluripotency of stem cells, MAPK signaling pathway, etc (Figure 3).

### Functional Enrichment Analysis of Differentially Expressed Genes

**FIGURE 4:**
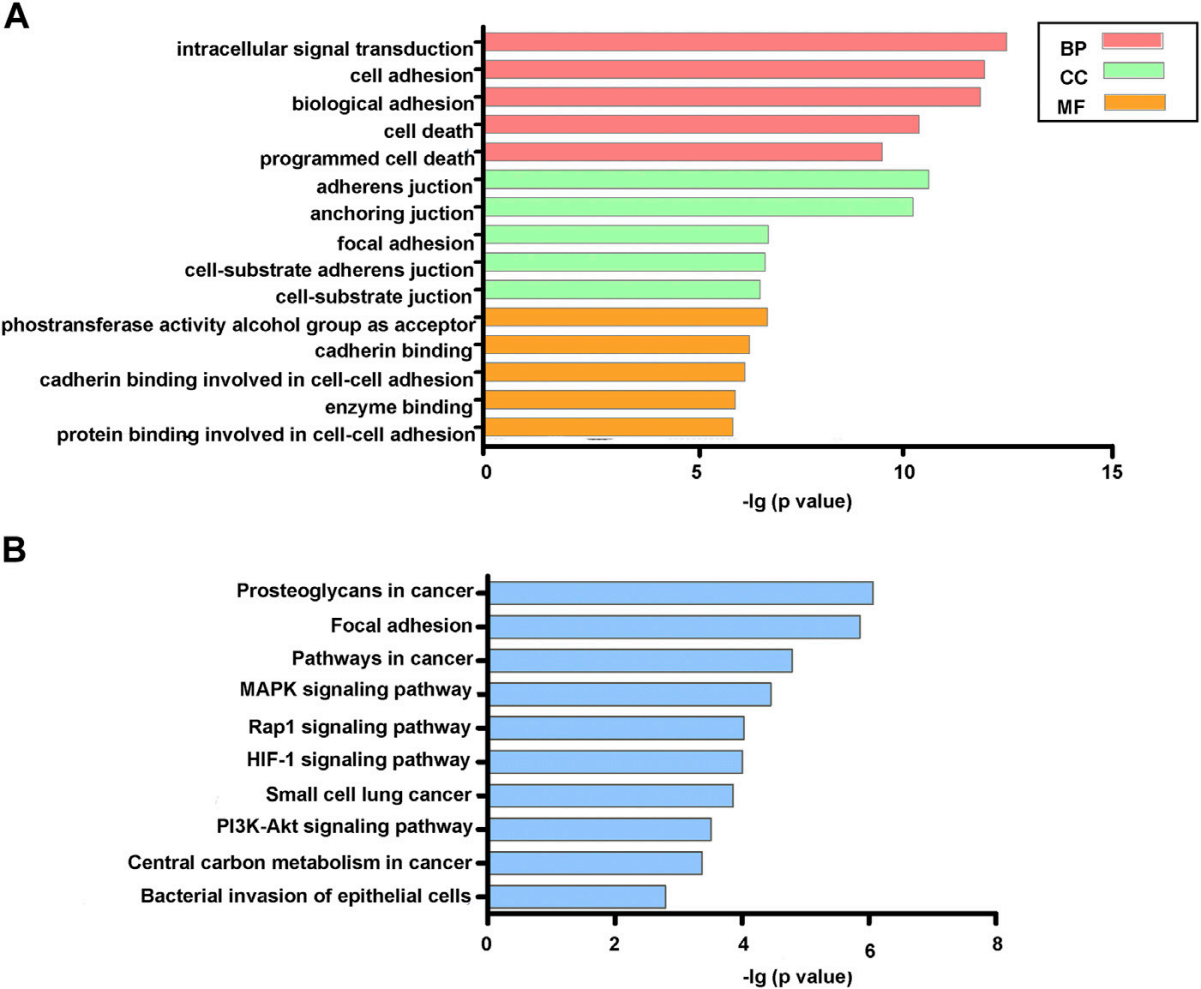
GO **(A)** and KEGG **(B)** functional enrichment analysis of DEGs.

As shown in Figure 4A, the final list of DEGs were mainly enriched in biological processes (BP), including intracellular signal transduction, cell and biological adhesion, and cell and programmed cell death. As for cellular components (CC), DEGs were significantly involved in adherens and anchoring junctions, and focal adhesion. For molecular function (MF), the significant participants included phosphotransferase activity alcohol group as acceptor and cadherin and enzyme binding. KEGG analysis indicated that DEGs were enriched in several tumorigenesis- and angiogenesis-related pathways such as proteoglycans in cancer, pathways in cancer, MAPK, Rap1, and HIF-1 signaling pathways (Figure 4B).

### Protein-Protein Interaction of Differentially Expressed Genes

**FIGURE 5:**
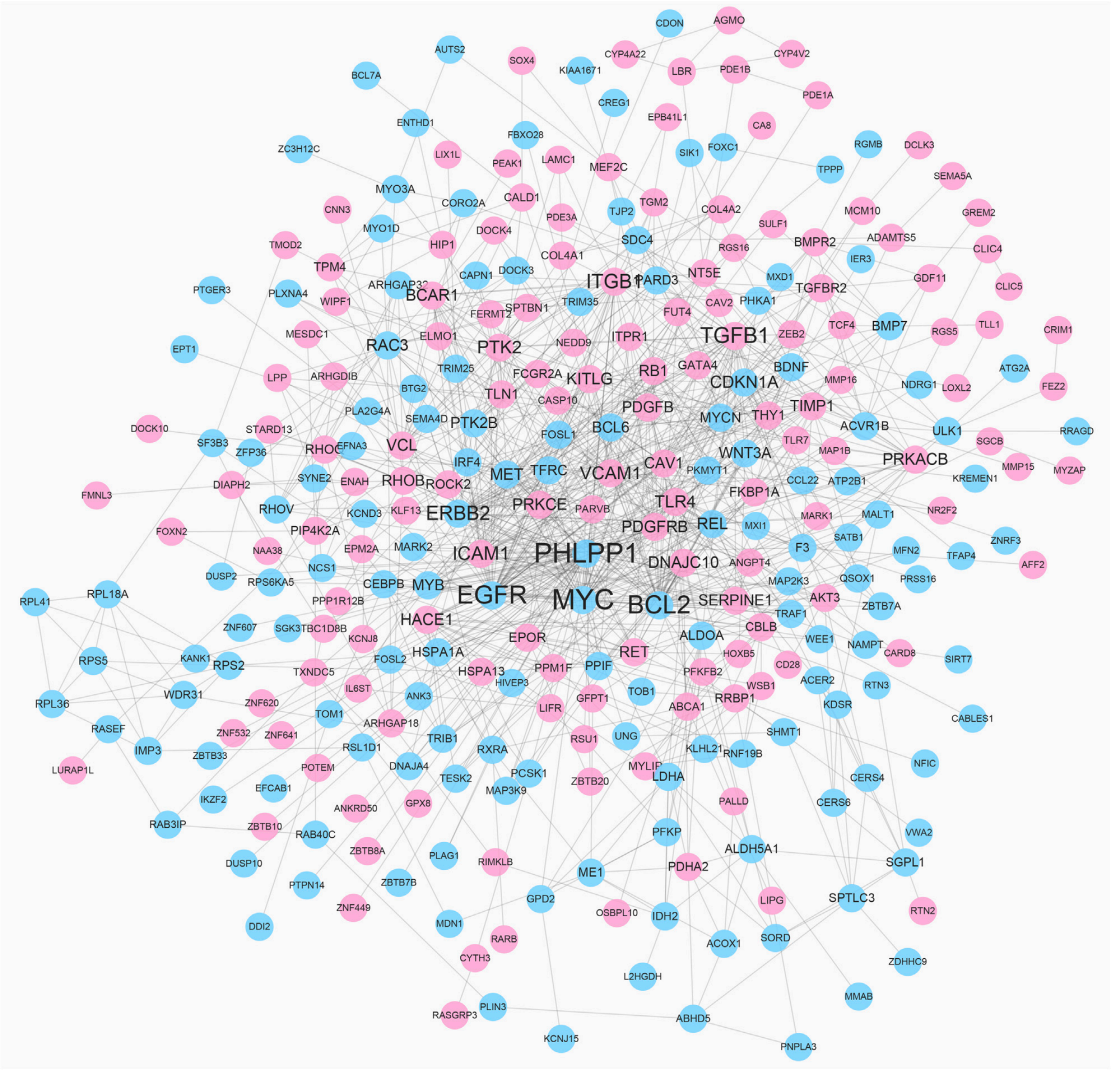
PPI network of DEGs. Red nodes represent up-regulated DEGs and blue nodes represent down-regulated DEGs. Nodes with higher degree are depicted with larger label size.

**FIGURE 6:**
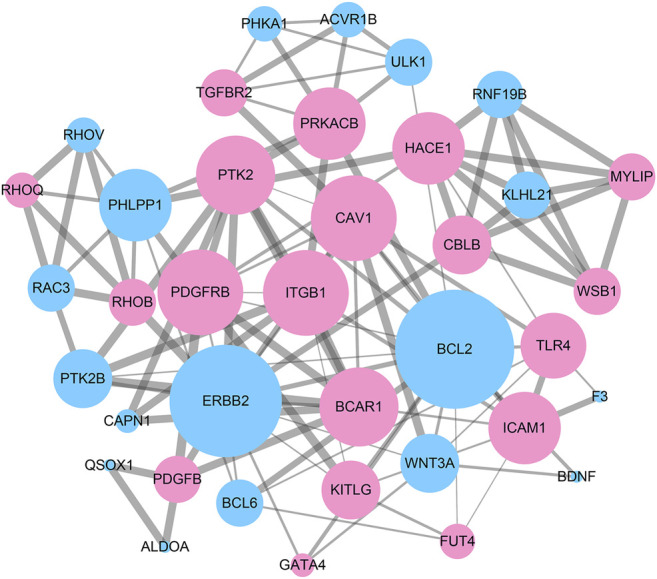
A sub-network module with the highest score in PPI network consisting of 37 nodes and 113 edges. Red nodes represent up-regulated DEGs and blue nodes represent down-regulated DEGs. Nodes with higher degree are performed with larger size. Edges with higher combined_score are depicted with thicker lines.

To reveal the interaction among DEGs, a PPI network was constructed using Cytoscape, which consisted of 315 nodes and 965 edges (Figure 5). The top 10 DEGs were selected as hub genes of IH based on interaction degree. These hub genes included the regulator gene (*MYC*), PH domain and leucine rich repeat protein phosphatase (*PHLPP1*)*,* B-cell lymphoma 2 (*BCL2*)*,* epidermal growth factor (*EGFR*), transforming growth factor beta 1 (*TGFB1*), integrin beta 1 (*ITGB1*), erb-b2 receptor tyrosine kinase (*ERBB2*), intracellular adhesion molecule 1 (*ICAM1*), protein tyrosine kinase 2 (*PTK2*), and vascular cell adhesion molecule 1 (*VCAM1*). A modular DEG analysis was performed with the plug-in MCODE of Cytoscape. A module consisting of 37 nodes and 113 edges was identified (Figure 6). Pathway analysis showed modular genes were significantly enriched in cancer pathways and focal adhesion pathway (Table 1).

**TABLE 1:** Functional enrichment analysis of DEGs in module.

Term	Description	Count	*p*-value	FDR
GO: 0051270	regulation of cellular component movement	18	1.24E-13	2.23E-10
GO: 0009966	regulation of signal transduction	25	2.11E-11	3.77E-08
GO: 0009966	positive regulation of cellular component movement	13	4.11E-11	7.36E-08
GO: 0006928	movement of cell or subcellular component	21	5.39E-11	9.66E-08
GO: 0006928	regulation of cell communication	25	1.92E-10	3.43E-07
KEGG: hsa05200	Pathways in cancer	12	1.56E-07	1.84–04
KEGG: hsa04510	Focal adhesion	9	1.01E-06	0.001
KEGG: hsa05205	Proteoglycans in cancer	8	1.08E-05	0.013

## Discussion

Proliferative IH may lead to several severe complications, which represent a great burden to patients and families. MicroRNAs have been demonstrated to act as vital regulatory factors in gene expression of various tumors (Sand et al., 2012; Sand et al., 2017). Although several studies have reported a few specific miRNAs function in IH (Strub et al., 2016; Li et al., 2017), the comprehensive miRNA-gene interaction network in IH remains largely unknown.

Our study showed the miRNA profile of IH and identified miR-519d-3p, miR-21-5p, miR-15a-5p, miR-34a-5p, miR-520a-3p, miR-520b, miR-520c-3p, miR-520d-3p, miR-424-5p, and miR-519a-3p as hub miRNAs in IH. The microarray results revealed that chromosome 19 miRNA cluster (C19MC) was significantly up-regulated in IH, which was consistent with the findings of Strub et al. (2016). Six of the hub miRNAs (miR-519d-3p, miR-520a-3p, miR-520b, miR-520c-3p, miR-520d-3p, and miR-519a-3p), which belong to C19MC, were also revealed to act as oncogenes or suppressors in different tumors (Xie and Sadovsky, 2016). The remaining four hub miRNAs have not been reported in IH previously, but they were all tumorigenesis-related. Previous findings have demonstrated miR-15a-5p accelerated progression of colorectal carcinoma by regulation of the mitochondrial uncoupling protein gene (*UCP2*) and the constitutive photomophogenesis nine gene (*COP9*) (de Groen et al., 2015). MiR-21-5p was reported to promote angiogenesis and selected as a biomarker for pancreatic cancer (Qu et al., 2017). Several studies have found that MiR-34a-5p regulated cell proliferation and apoptosis in osteosarcoma (Pu et al., 2017). Over-expression of miR-424-5p promoted cell proliferation and inhibited Smad3 expression through the TGF-beta pathway in gastric cancer (Wei et al., 2017). Pathway analysis of the hub miRNAs indicated that IH pathogenesis was associated with the TGF-beta signaling pathway, signaling pathways regulating pluripotency of stem cells, and the MAPK signaling pathway. Previous reports have demonstrated that proliferation and differentiation of hemangioma-derived stem cells (HemSC) dominated IH-genesis. Inhibition of MAPK pathway-enhanced HemSC proliferation and viability has been implicated in IH genesis (Munabi et al., 2016). The TGF-beta pathway has been reported to play a key role in endothelial mesenchymal transition in IH (Lou, 2017).

According to our study, a total of 475 DEGs were identified in IH, including 228 up-regulated and 247 down-regulated genes. GO analysis indicated DEGs were closely related to intracellular signal transduction pathways, cell adhesion, and cell death. KEGG analysis showed DEGs were mainly involved in the MAPK, Rap1, and HIF-1 pathways. Substantial evidence has suggested that IH proliferation was extensively associated with the HIF-1-VEGF angiogenic axis (Chim et al., 2012). The hub genes identified in IH included MYC, PHLPP1, BCL2, EGFR, TGFB1, ITGB1, ERBB2, ICAM1, PTK2, and VCAM1. A previous study indicated MYC, a stem cell marker, was highly expressed in IH endothelial cells, which supports the HemSC origin hypothesis (Spock et al., 2015). PHLPP1 may act as tumor suppressor and AKT phosphorylation regulator in several kinds of tumors (Vera et al., 2015). BCL2 is an important cellular apoptosis regulator in various tumors through interaction with BCL2 family proteins (Watson et al., 2017). Previous studies manifested that over expression of EGFR promoted cell proliferation and angiogenesis by activating MAPK and AKT/PI3K pathway (Wee and Wang, 2017). A key member of the EGFR family, ERBB2, has been elucidated in regulating development of breast and gastric cancers (Dupouy et al., 2016). It has been proposed that TGFB1 can be induced by hypoxia and controlled growth and differentiation of epithelial cells (Furuta et al., 2015). ITGB1 is an oncogene that inhibits cell adhesion and enhances cell migration and invasion (Klahan et al., 2016). In addition, ICAM1 and VCAM1, which are typically expressed in vascular endothelium, were both up-regulated in IH. The last hub gene is PTK2, which plays an important role in cell migration. In IH, the miR-130a inhibitor restrained cell invasion and migration by suppressing focal adhesion kinase signaling (Gao et al., 2017).

In conclusion, we identified the miRNA profile of IH and constructed DEMs-DEGs regulating network by integration of miRNA and mRNA profiles. Several hub miRNAs and genes of IH were identified by bioinformatics, and the key pathways, which were closely related to tumorigenesis and angiogenesis, were elucidated. This study should be beneficial to comprehension of IH pathogenesis and may provide latent targets for IH diagnosis and therapy. Nevertheless, further pathological mechanism and clinical studies containing large samples are required in the future.

## Data Availability

The datasets presented in this study can be found in online repositories. The names of the repository/repositories and accession number(s) can be found in the article/Supplementary Material.

## Supplementary Material

Supplementary Figure S1 The workflow of the methodology used in the study. miRNA(s), micro-RNA(s); IH, infantile hemangioma; DEG, differentially expressed genes between IH and normal tissues; DEM, differentially expressed miRNAs between IH and normal tissues.
