# Supplementary figures and images for: Identification of Key microRNAs and Genes in Infantile Hemangiomas
Source: Front Genet. 2022 Mar 11;13:766561. doi: 10.3389/fgene.2022.766561 (PMC8963821; doi:10.3389/fgene.2022.766561)

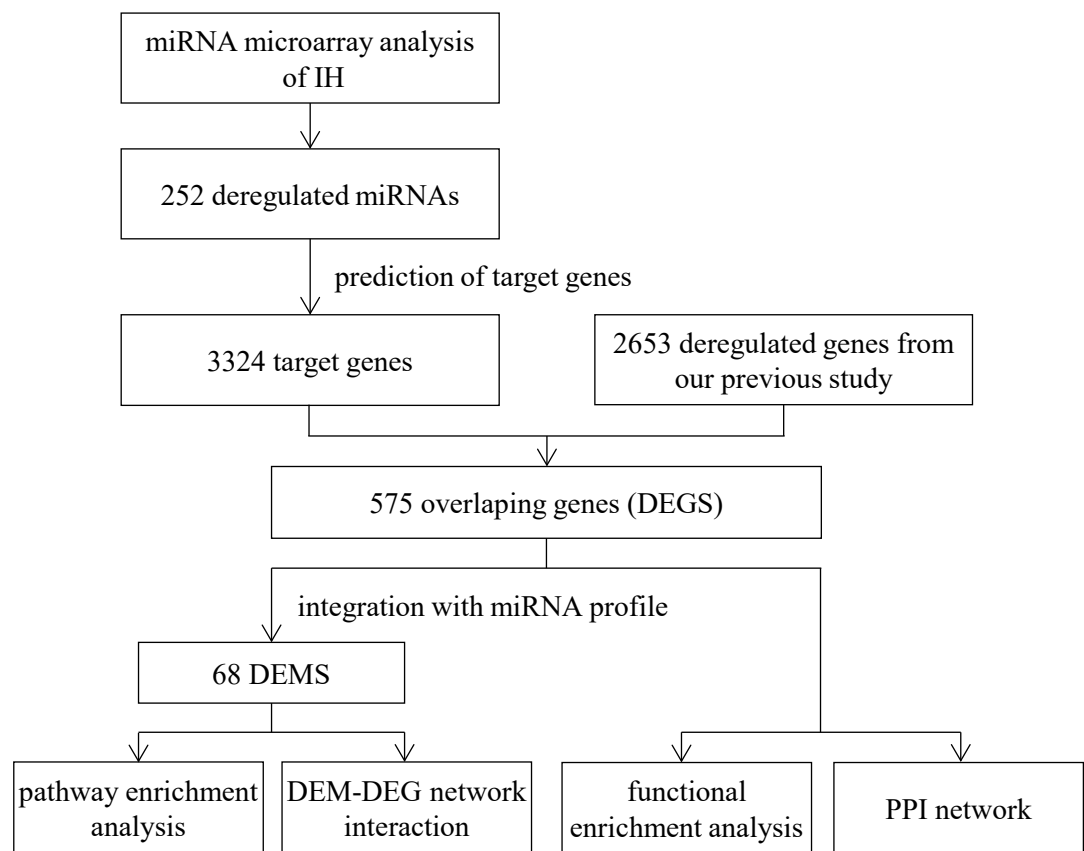

Supplement: Supplementary file 1 [file DataSheet1.PDF]
